# A Resistance Mechanism in Non-*mcr* Colistin-Resistant Escherichia coli in Taiwan: R81H Substitution in PmrA Is an Independent Factor Contributing to Colistin Resistance

**DOI:** 10.1128/spectrum.00022-21

**Published:** 2021-07-14

**Authors:** Ching-Hsun Wang, L. Kristopher Siu, Feng-Yee Chang, Sheng-Kang Chiu, Jung-Chung Lin

**Affiliations:** a Graduate Institute of Medical Sciences, National Defense Medical Center, Taipei, Taiwan; b Institute of Infectious Diseases and Vaccinology, National Health Research Institutes, Zhunan, Miaoli, Taiwan; c Division of Infectious Diseases and Tropical Medicine, Department of Internal Medicine, Tri-Service General Hospital, National Defense Medical Center, Taipei, Taiwan; Houston Methodist Hospital

**Keywords:** colistin, resistance, *E. coli*, Taiwan, ST131, ST1193, non-*mcr*, independent, chromosome

## Abstract

Colistin resistance due to the *mcr*-type genes in Escherichia coli is well characterized. In order to study the resistance mechanism in *mcr*-negative colistin-resistant E. coli, strains were selected from a nationwide antimicrobial resistance surveillance program in Taiwan for further investigation. A total of 11 *mcr*-negative colistin-resistant isolates among 7,942 (0.1%) clinical E. coli isolates were identified between 2008 and 2018. Their prevalence was low and remained stable during the study period. Since 2012, ST131 and ST1193 clones with multiple drug-resistant phenotypes have emerged. All resistant strains displayed higher expression levels of the operons *pmrHFIJKLM* and *pmrCAB* than the control MG1655 strain. Although several amino acid substitutions were identified in PmrA or PmrB, only R81H in PmrA was associated with overexpression of *pmrHFIJKLM* and colistin resistance. The effect of substitution R81H in PmrA in colistin resistance was confirmed by complementation experiments. Although some strains harbored substitutions in PmrB, the identified mutations in *pmrB* did not contribute to colistin resistance. In conclusion, the amino acid substitution R81H in PmrA is an independent factor contributing to colistin resistance in non-*mcr* E. coli.

**IMPORTANCE** The molecular epidemiology and resistance mechanisms of *mcr*-negative colistin-resistant E. coli are not well described. In this study, a total of 11 *mcr*-negative colistin-resistant E. coli isolates were selected from a nationwide antimicrobial resistance surveillance program in Taiwan for further investigation. We determined the resistance mechanism of non-*mcr* colistin-resistant strains using gene knockout and complementation experiments. We observed the occurrence of the global multiple-drug-resistant E. coli clones ST131 and ST1193 starting in 2012. Moreover, for the first time, we proved that the amino acid substitution R81H in PmrA is an independent factor contributing to colistin resistance in non-*mcr* E. coli. The study results helped to gain an insight into the diversity and complexity of chromosome-encoded colistin resistance in E. coli.

## INTRODUCTION

Colistin, a cationic antimicrobial peptide targeting lipopolysaccharide (LPS) of Gram-negative bacteria, has been considered one of the last-resort drugs to treat carbapenem-resistant *Enterobacteriaceae* (CRE) infections (1). Due to the increased use of colistin, reports of colistin resistance in *Enterobacteriaceae* have emerged worldwide (2). A major acquired resistance mechanism to colistin involves plasmid- or chromosome-mediated modifications of LPS. LPS is a main constituent of the outer membrane of Gram-negative bacteria. Alteration of the LPS could reduce the electrostatic affinity between LPS and positively charged colistin, thereby triggering the bacteria to exhibit colistin resistance (3).

Plasmid-mediated transferable colistin resistance occurs through mobile colistin resistance (*mcr*) genes, which were initially discovered in China and have already spread worldwide (4, 5). This gene encodes phosphoethanolamine transferase, which modifies lipid A of LPS. Therefore, LPS affinity for colistin is reduced and thereby contributes to colistin resistance. In contrast, LPS modification in *Enterobacteriaceae* could also be mediated by a chromosome-encoded mechanism linked to acquisition of mutations in genes involving regulation of the PmrAB and PhoPQ two-component system (TCS) connected by *pmrD* (3). Studies of chromosome-encoded colistin mechanisms were extensively reported in Salmonella and Klebsiella pneumoniae, revealing that mutated regulatory genes, including *pmrAB*, *phoPQ*, *crrB*, and *mgrB*, will constitutively activate the PmrAB or PhoPQ TCS, resulting in increased downstream *pmrHFIJKLM* and *pmrCAB* operon expression, which is responsible for LPS modification, eventually resulting in colistin resistance (6–12). Although the mutated *pmrD* contributing to colistin resistance has not been observed, overexpression of the *pmrD* gene conferring colistin resistance has been reported in Salmonella enterica serovar Typhimurium (13). Studies on chromosomal colistin resistance in Escherichia coli, another important pathogen among the *Enterobacteriaceae*, showed diverse results. Previous studies on chromosomal colistin-resistant E. coli revealed that contributory mutated genes to colistin resistance were confined to *pmrAB* only, whereas mutations in *mgrB* and *phoPQ* have not been reported yet (14–18). The difference may be related to an increased rate of PmrA dephosphorylation in E. coli compared to other species of *Enterobacteriaceae*. This could neutralize the activating effects of PhoPQ TCS regulated by *phoPQ* and *mgrB* in E. coli (19). As a consequence, mutated *phoPQ* or *mgrB* leading to PhoPQ TCS upregulation could not influence PmrAB TCS via the connector protein PmrD. Presently, limited studies with few E. coli isolates describing chromosomal colistin resistance mechanisms using a formal experimental method have been reported. Furthermore, fewer data are available on the estimated prevalence and molecular epidemiology of chromosomal colistin-resistant E. coli.

Taiwan Surveillance of Antimicrobial Resistance (TSAR) is a nationwide program used to survey antimicrobial resistance among organisms of clinical importance in Taiwan (20). In a previous study, Kuo et al. described the prevalence of *mcr*-*1* in E. coli and the molecular characteristics of isolates with this gene in Taiwan from the TSAR program (21). To further characterize the chromosomal colistin resistance mechanism of E. coli, we studied a collection of E. coli isolates from TSAR with chromosome-borne colistin resistance genes for their molecular epidemiology and resistance mechanisms.

## RESULTS

### Prevalence and characteristics of *mcr*-negative colistin resistance in E. coli.

In total, 7,942 nonduplicate clinical E. coli isolates were studied, including 1,136, 1,752, 1,701, 1,650, and 1,703 from 2008 to 2010, 2010 to 2012, 2012 to 2014, 2014 to 2016, and 2016 to 2018, respectively. Initially, a total of 66 isolates had colistin MICs of >2 mg/liter during the study period. Fourteen isolates with *mcr-1* were reported before 2014, and 41 isolates that harbored *mcr*-*1* were reported after this, with 13 (0.9%) from 2014 to 2016 and 28 (1.7%) from 2016 to 2018. After *mcr*-*1*-carrying isolates were excluded, a total of 11 isolates were identified. Further investigation revealed that 11 isolates were negative for *mcr*-*2* to *mcr*-*9*. Moreover, conjugation assays using these 11 colistin-resistant E. coli isolates as donors and E. coli J53 as the recipient revealed no transconjugants from Mueller-Hinton agar plates containing colistin (4 mg/liter) and azide (500 mg/liter). Results indicated that colistin resistance mechanisms from the 11 E. coli isolates may be mediated by chromosomes and not by plasmids. Thereafter, we designated these isolates TSAREC01, TSAREC02, TSAREC03, TSAREC04, TSAREC05, TSAREC06, TSAREC07, TSAREC08, TSAREC10, TSAREC37, and TSAREC41.

**FIG 1:**
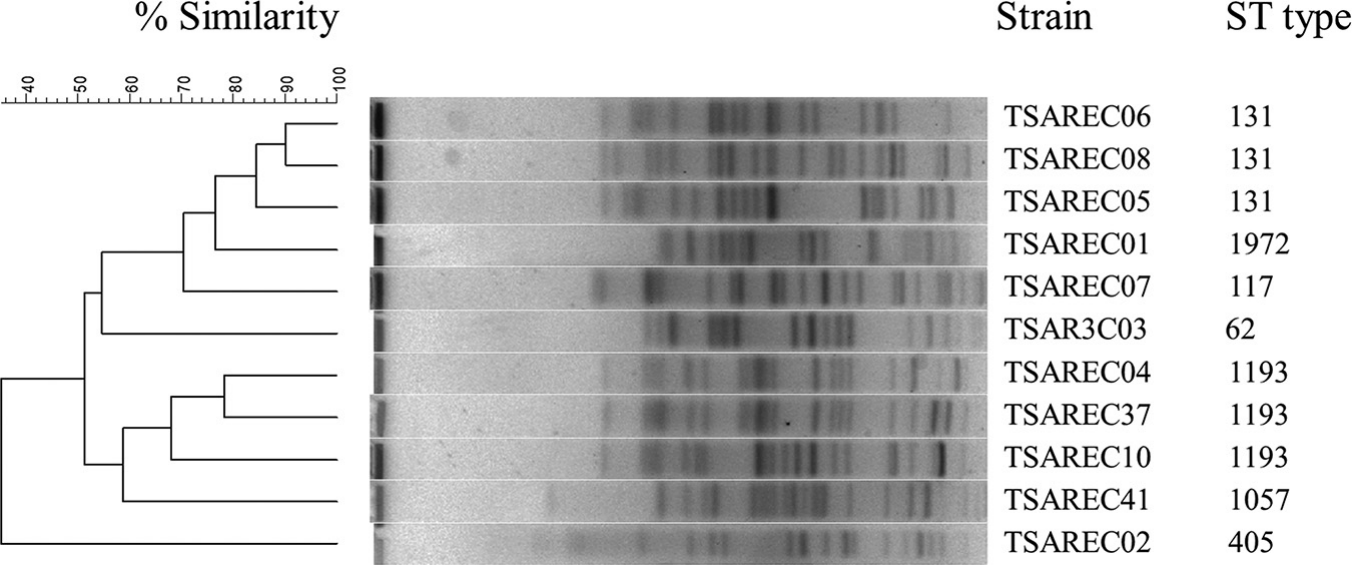
Dendrogram analysis and virtual gel images based on PFGE results for XbaI-digested genomic DNA.

Table 1 summarizes characteristics of the identified strains. The 11 colistin-resistant E. coli isolates were found in urine (six), blood (three), abscess (one), and sputum (one). These isolates were obtained from hospitals located in all four regions of Taiwan. Six of these isolates were recovered from outpatients, whereas five were from inpatients. The MIC of colistin for the 11 strains ranged from 8 to 16 mg/liter, and 3 strains harbored CTX-M-type β-lactamase (*bla*_CTX-M-G1_ for TSAREC07, *bla*_CTX-M-G9_ for TSAREC08 and TSAREC37). From 2008 to 2012, the isolates belonged to diverse sequence types (STs), but ST131 and ST1193 have emerged as the major STs among isolates since 2012. Pulsed-field gel electrophoresis (PFGE) revealed that one cluster of ST131 E. coli strains shared ≥80% similarity in PFGE pattern, while other strains belonged to diverse pulsotypes (Fig. 1).

**TABLE 1:** Characteristics of colistin-resistant E. coli strains in the present study^*a*^

Strain	Period of isolation	Region of Taiwan	Hospital level	Hospital location of isolation	Specimen source	MLST (ST)	Colistin MIC (mg/liter)^*b*^	Additional ESBL genes
TSAREC01	2008–2010	Central	MC	ICU	Abscess	1972	8	
TSAREC02	2008–2010	Central	RH	Non-ICU	Urine	405	16	
TSAREC03	2010–2012	Northern	RH	OPD	Blood	62	8	
TSAREC04	2012–2014	Northern	MC	OPD	Urine	1193	8	
TSAREC05	2012–2014	Central	RH	Non-ICU	Sputum	131	16	
TSAREC06	2012–2014	Central	RH	OPD	Blood	131	8	
TSAREC07	2014–2016	Southern	MC	Non-ICU	Urine	117	8	CTX-M-1 group
TSAREC08	2014–2016	Eastern	RH	ICU	Urine	131	8	CTX-M-9 group
TSAREC10	2014–2016	Central	RH	OPD	Urine	1193	16	
TSAREC37	2016–2018	Southern	RH	OPD	Urine	1193	16	CTX-M-9 group
TSAREC41	2016–2018	Central	MC	OPD	Blood	1057	8	

a MC, medical center; RC, regional hospital; ICU, intensive care unit; OPD, outpatient department; ESBL, extended-spectrum β-lactamases.

b MICs of colistin were determined by the broth microdilution method.

MICs of antibiotics other than colistin for the 11 E. coli strains are shown in Table 2. Rates of resistance to other antibiotics were as follows: 45.4% to gentamicin, 72.7% to ampicillin and ciprofloxacin, 54.5% to levofloxacin and cefazolin, 36.4% to ceftriaxone and trimethoprim-sulfamethoxazole, and 27.3% to ceftazidime. No isolates were resistant to cefepime, amikacin, piperacillin-tazobactam, or imipenem/cilastatin.

**TABLE 2:** MICs of antibiotics against 11 colistin-resistant E. coli strains^*a*^

Strain	MIC (mg/liter)											
	CFZ	AMP	TZP	CRO	CAZ	FEP	IMP	GEM	AMK	LVX	CIP	SXT^*b*^
TSAREC01	8	>32	<4	<1	<1	<1	<0.25	<1	<2	<0.12	<0.25	<20
TSAREC02	32	>32	16	<1	<1	<1	<0.25	>16	<2	1	1	>320
TSAREC03	<4	>32	<4	<1	<1	<1	<0.25	>16	<2	0.5	<0.25	<20
TSAREC04	<4	<2	<4	<1	<1	<1	<0.25	<1	<2	>8	>4	<20
TSAREC05	>64	>32	32	16	16	<1	<0.25	>16	4	>8	>4	>320
TSAREC06	<4	4	<4	<1	<1	<1	<0.25	<1	<2	>8	>4	<20
TSAREC07	>64	>32	<4	>64	16	4	<0.25	<1	<2	1	1	>320
TSAREC08	>64	>32	16	>64	>64	4	1	<1	<2	>8	>4	>320
TSAREC10	<4	>32	<4	<1	<1	<1	<0.25	>16	<2	>8	>4	<20
TSAREC37	>64	>32	64	>64	2	2	<0.25	>16	<2	>8	>4	<20
TSAREC41	<4	<2	<4	<1	<1	<1	<0.25	<1	<2	0.5	<0.25	<20

a CFZ, cefazolin; AMP, ampicillin; TZP, piperacillin-tazobactam; CRO, ceftriaxone; CAZ, ceftazidime; FEP, cefepime; IMP, imipenem-cilastatin; GEM, gentamicin; AMK, amikacin; LVX, levofloxacin; CIP, ciprofloxacin; SXT, trimethoprim-sulfamethoxazole.

b SXT MICs are reported as the sum of trimethoprim and sulfamethoxazole MICs, which are present in a ratio of 1:19, according to the Vitek 2 automated system. The SXT resistance breakpoint in this system is >80 mg/liter.

### Expression levels of the *pmrD* gene, the *pmrCAB* operon, and the *pmrHFIJKLM* operon in colistin-resistant E. coli strains.

Expression levels of *pmrD*, *pmrC*, and *pmrK* genes in the identified colistin-resistant E. coli strains were compared with those in the wild-type MG1655 strain (Table 3). All colistin-resistant strains displayed significantly increased expression levels for both *pmrC* and *pmrK* genes. For *pmrD* expression levels, no increased levels among all colistin-resistant strains were observed.

**TABLE 3:** Amino acid changes in PmrA/PmrB^*a*^ and gene expression levels of colistin-resistant E. coli strains

Strain	Substitution(s) in^*b*^:		Relative expression level (mean ± SD)^*c*^		
	PmrA	PmrB	*pmrD*	*pmrK*	*pmrC*
TSAREC01	R81H		1.20 ± 0.39	115.41 ± 44.95	143.12 ± 50.09
TSAREC02		G206R, Y222H	0.78 ± 0.35	103.14 ± 38.34	168.64 ± 63.00
TSAREC03		M11, L14P, P178S, T235N	0.53 ± 0.22	123.54 ± 49.64	720.94 ± 343.66
TSAREC04	R81H		0.47 ± 0.15	61.35 ± 22.42	101.36 ± 24.14
TSAREC05		P94L	0.22 ± 0.06	64.79 ± 36.92	148.06 ± 89.43
TSAREC06		G19E	0.44 ± 0.17	45.01 ± 7.15	103.35 ± 41.94
TSAREC07		P94L	0.63 ± 0.21	99.80 ± 40.17	295.63 ± 117.03
TSAREC08		L194P	0.38 ± 0.20	94.36 ± 41.61	201.4 ± 67.89
TSAREC10		L98R	0.52 ± 0.12	54.44 ± 16.91	216.15 ± 47.24
TSAREC37		L27R	0.65 ± 0.14	25.19 ± 8.06	122.06 ± 52.40
TSAREC41	R81H		0.36 ± 0.07	59.50 ± 18.18	117.67 ± 39.61

a Amino acid substitutions found only in colistin-resistant E. coli isolates after alignment with 8 clinical colistin susceptible E. coli strains (ECS01 to ECS08) and MG1655.

b The one-letter designations for amino acids are used.

c Expression levels of *pmrD*, *pmrC*, and *pmrK* genes (presented as fold change) were normalized against the value for MG1655. Values are means and standard deviations from four independent experiments.

### Amino acid substitutions in PmrAB and PmrD in colistin-resistant E. coli strains.

Sequence comparisons of *pmrA*, *pmrB*, and *pmrD* genes in MG1655 with those in eight colistin-susceptible strains (ECS01 to ECS08) were performed. All amino acid substitutions in PmrD among colistin-resistant E. coli strains relative to MG1655 were also observed in colistin-susceptible strains. The nucleotide variations of PmrA and PmrB that produce amino acid substitutions only in colistin-resistant strains after comparison are shown in Table 3. The R81H substitution in PmrA and G19E, L14P, L27R, L194P, L98R, and P94L in PmrB were predicted to affect the function of proteins encoded by these genes after analysis using Protein Variation Effect Analyzer (PROVEAN) and Sorting Intolerant From Tolerant (SIFT) software.

### Role of amino acid substitutions in PmrAB in colistin-resistant strains.

**FIG 2:**
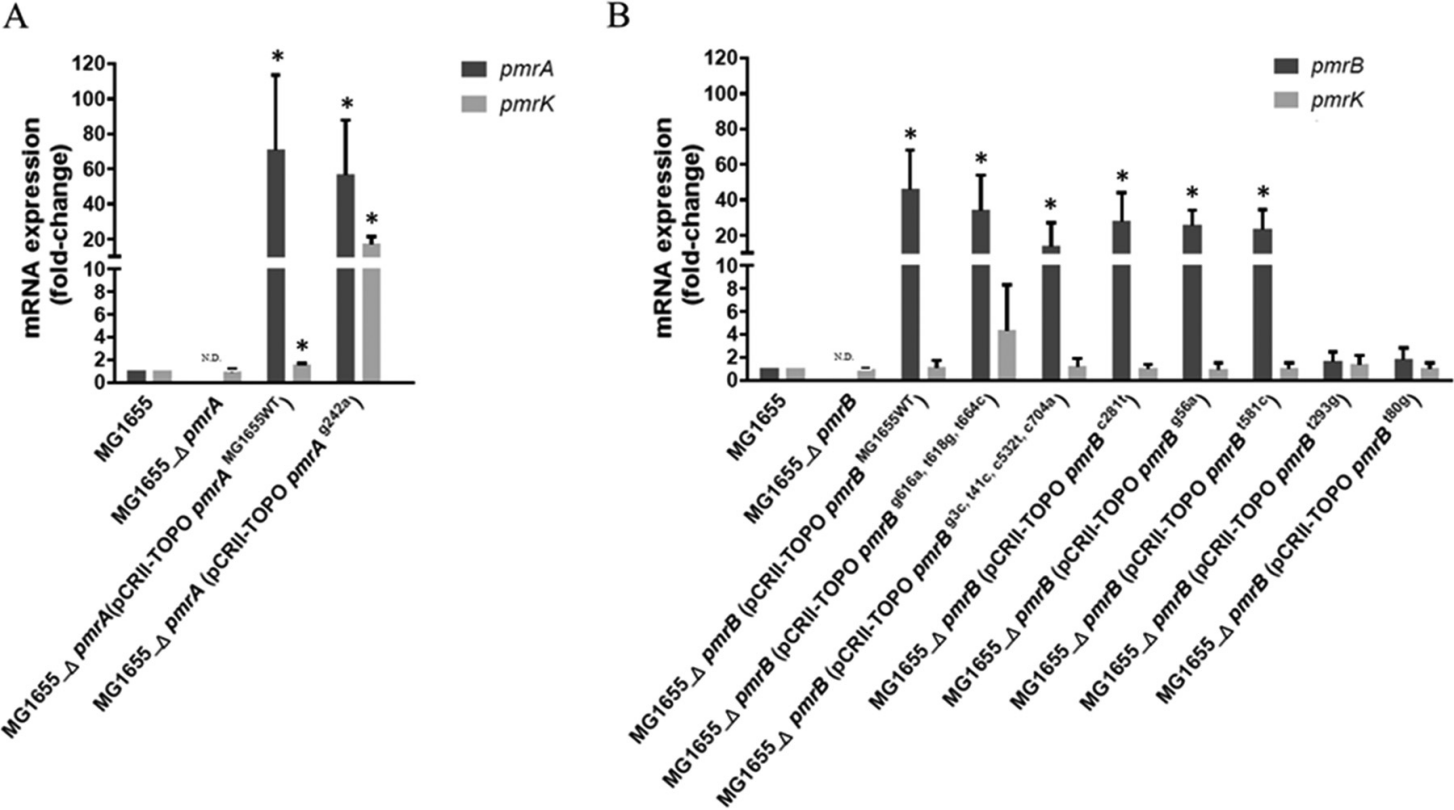
mRNA expression levels of *pmrA* and *pmrK* (A) and *pmrB* and *pmrK* (B) in MG1655-derived strains. Values are relative expression levels (fold change) normalized against MG1655 and determined via quantitative PCR. The values are means and standard deviations from four independent experiments. N.D., not detected. *, significant difference compared to MG1655 (*P < *0.05).

The roles of the alterations of PmrA and PmrB detected in colistin-resistant strains were further investigated by complementation experiments. Following transformation with a recombinant plasmid containing a mutated *pmrA* allele, the complemented strain MG1655_Δ*pmrA*(pCRII-TOPO*pmrA*^g242a^) exhibited resistance to colistin (MIC = 8 mg/liter) and presented increased *pmrK* expression with significance (relative change, 17.12 ± 4.23-fold relative to MG1655) (Table 4 and Fig. 2A). Moreover, another complemented strain, MG1655_Δ*pmrA*(pCRII-TOPO*pmrA*^WT^), was still susceptible to colistin (MIC = 0.5 mg/liter) after transformation of wild-type PmrA, and the *pmrK* expression level was 1.52 ± 0.18-fold relative to MG1655. Collectively, these results supported the idea that a mutated *pmrA* allele encoding the R81H substitution in PmrA conferred colistin resistance in E. coli.

**TABLE 4:** Results of complementation experiments with different E. coli MG1655-derived strains

Strain	Colistin MIC (μg/ml)	Relative expression level (mean ± SD)^*a*^		
		*pmrA*	*pmrB*	*pmrK*
MG1655	0.5	1	1	1
MG1655_Δ*pmrA*	0.5	<0.001		0.91 ± 0.34
MG1655_Δ*pmrB*	0.5		<0.001	0.91 ± 0.13
MG1655_Δ*pmrA* (pCRII-TOPO*pmrA*^MG1655WT^)	0.5	59.91 ± 30.88		1.52 ± 0.18
MG1655_Δ*pmrB* (pCRII-TOPO*pmrB*^MG1655WT^)	0.5		45.79 ± 22.30	1.12 ± 0.63
MG1655_Δ*pmrA* (pCRII-TOPO*pmrA*^g242a^)	8	70.80 ± 42.77		17.12 ± 4.23
MG1655_Δ*pmrB* (pCRII-TOPO*pmrB*^g616a, t618g, t664c^)	0.5		34.27 ± 19.65	4.32 ± 3.99
MG1655_Δ*pmrB* (pCRII-TOPO*pmrB*^g3c, t41c, c532t, c704a^)	0.5		14.01 ± 13.05	1.24 ± 0.68
MG1655_Δ*pmrB* (pCRII-TOPO*pmrB*^c281t^)	0.5		28.19 ± 15.74	1.01 ± 0.39
MG1655_Δ*pmrB* (pCRII-TOPO*pmrB*^g56a^)	0.5		25.86 ± 8.16	0.94 ± 0.56
MG1655_Δ*pmrB* (pCRII-TOPO*pmrB*^t581c^)	0.5		23.24 ± 11.13	1.03 ± 0.48
MG1655_Δ*pmrB* (pCRII-TOPO*pmrB*^t293g^)	0.5		1.68 ± 0.81	1.38 ± 0.79
MG1655_Δ*pmrB* (pCRII-TOPO*pmrB*^t80g^)	0.5		1.83 ± 1.00	1.03 ± 0.48

a Expression levels of *pmrA*, *pmrB*, and *pmrK* (presented as fold change) were normalized against the value for MG1655. Values are means and standard deviations from four independent experiments.

Complemented strains of MG1655_Δ*pmrB* transformed with different mutated PmrB proteins are shown in Table 4. Expression levels of *pmrB* among different complemented strains varied, but all complemented strains were still susceptible to colistin (MIC = 0.5 mg/liter). The *pmrK* mRNA expression levels of each of the complemented strains revealed no significant change compared to that of MG1655 (Table 4 and Fig. 2). In another complementation experiment, all colistin-resistant strains with mutated *pmrB* (TSAREC02, TSAREC03, TSAREC05, TSAREC06, TSAREC08, TSAREC10, and TSAREC37) were complemented with the wild-type *pmrB* from MG1655. No change in the MIC of colistin was observed among the complemented strains. Taken together, these results suggest that the mutated *pmrB* gene identified in these colistin-resistant E. coli strains may not contribute to colistin resistance.

## DISCUSSION

In the present study, we describe the molecular epidemiology and resistance mechanisms of *mcr*-negative colistin-resistant E. coli in Taiwan. The prevalence of *mcr*-negative colistin-resistant E. coli during the study period remained low (<1%) with no interval change. The results were consistent with those reported in previous studies in China (22), Japan (23), and the Netherlands (16). In contrast, one study by Bourrel et al. revealed higher prevalence (11.9%) among inpatients of six university hospitals in France (17). In this study, the results were obtained from selected inpatients with risk factors for multiple drug resistance in the ICU or hospital admissions, rather than from the general inpatient population. Therefore, the prevalence estimation may be biased. Notably, we observed the emergence of globally disseminated multiple-drug-resistant clones ST131 and ST1193 among colistin-resistant E. coli strains since 2012. Colistin-resistant E. coli isolates of ST131 have been reported before; however, another new global virulent clone, ST1193, was observed for the first time in the current study (16, 23). E. coli ST131 and ST1193 are usually associated with resistance to fluoroquinolone and extended spectrum cephalosporins and widely cause epidemics among humans (24, 25). Concomitant colistin resistance in such multiple-drug-resistant clones would further restrict the current limited therapeutic options available to clinicians and endanger infected patients. Although, at present, this prevalence is relatively low, continued surveillance is mandatory.

All colistin-resistant strains in this study displayed higher *pmrK* and *pmrC* mRNA expression levels than MG1655, thereby indicating that increased expression of *pmrHFIJKLM* and *pmrCAB* operons under the control of the PmrAB system, leading to LPS modification, contributes to colistin resistance in E. coli. Moreover, none of the colistin-resistant strains displayed increased *pmrD* expression levels compared to MG1655. The results indicated that the colistin resistance mechanism of the studied E. coli strains may not be from *pmrD* overexpression, as previously described for *S.* Typhimurium (13).

In the present study, we confirmed that the R81H substitution in PmrA is an independent factor conferring a colistin resistance phenotype in TSAREC01, TSAREC04, and TSAREC41, experimentally. The R81H substitution in PmrA contributes to colistin resistance exclusively in S. enterica serovar Typhimurium from spontaneous mutants under laboratory condition and from clinical isolates (9, 26). The R81H substitution in the N-terminal response regulator domain of PmrA could protect phosphorylated-PmrA, the active form of PmrA, from PmrB-promoted dephosphorylation. Therefore, phosphorylated PmrA with higher binding affinity on target promoters than *pmrA* could result in increased expression of PmrA-activated genes, including the *pmrCAB* and *pmrHFIJKLM* operons, contributing to colistin resistance (27). This is the first study to observe and experimentally confirm the role of PmrA (R81H) in colistin resistance in clinical E. coli. In addition to the three strains studied, similar substitutions in PmrA (R81S and R81L) were observed in E. coli strains from different countries, although not this was not experimentally confirmed (17, 28). This suggested that this position in PmrA was critical for clinical colistin-resistant E. coli isolates.

In addition to PmrA substitutions in TSAREC01, TSAREC04, and TSAREC41, the remaining colistin-resistant strains have several substitutions in PmrB, and some of them were predicted to be deleterious for protein function according to two *in silico*-based software. programs. However, further investigation via complementation experiments indicated that all identified mutated PmrB proteins do not contribute to colistin resistance. Presumably, these strains have become resistant to colistin through other, unknown mutated factors involving PmrAB TCS regulation or a novel dysregulated pathway, thus bypassing the PmrAB TCS, which activated downstream *pmrCAB* and *pmrHFIJKLM* operon expression, eventually leading to colistin resistance. Further studies elucidating the mechanism of resistance to colistin in the remaining strains are under way.

In conclusion, occurrence of the global spread of clones ST131 and ST1193 among colistin-resistant E. coli strains since 2014 from a nationwide program is alarming and requires continued surveillance. The substitution R81H in PmrA conferring colistin resistance in *mcr*-negative E. coli was identified and experimentally confirmed in the present study.

## MATERIALS AND METHODS

### Bacterial strains and plasmids.

The colistin-resistant E. coli strains studied in this work were initially identified using Sensititre panels (Trek Diagnostics, England) from the biennial TSAR program from 2008 to 2018 (21). The E. coli strains, plasmids, and primers used in this study are listed in Tables S1, S2, and S3, respectively.

### Antimicrobial susceptibility and extended-spectrum-β-lactamase (ESBL) gene detection.

The MICs of different antibiotics for identified E. coli isolates were determined using the Vitek 2 (bioMérieux, France) system, with the exception of colistin. For determining MICs of colistin, the broth microdilution method was performed according to CLSI guidelines. The breakpoints of all tested antibiotics were interpreted according to guidelines from the CLSI with the exception of tigecycline and colistin, which were interpreted according to the EUCAST guidelines (http://www.eucast.org/clinical_breakpoints). E. coli ATCC 25922 was used as a quality control strain. Furthermore, CTX-M-type β-lactamase genes in identified colistin-resistant strains were detected by PCR amplification as described before (29).

### Detection of *mcr* genes and conjugation assay.

Possession of *mcr*-*1* to -*9* was detected via PCR in identified colistin-resistant E. coli strains. Moreover, conjugation experiments were performed using each of the colistin-resistant E. coli isolates as donors and E. coli J53, which is resistant to sodium azide, as the recipient strain. Transconjugants were selected on Mueller-Hinton agar containing sodium azide (500 mg/liter) plus colistin (4 mg/liter). The colistin-resistant E. coli strain with *mcr*-*1* EC909 described before was used as a positive control (30).

### Multilocus sequence typing (MLST) and genetic relatedness analysis.

The sequences of seven loci (*adk*, *fumC*, *gyrB*, *icd*, *mdh*, *purA*, and *recA*) were amplified using PCR, and STs were determined by sequence alignment using the Achtman scheme available at https://pubmlst.org/escherichia/. Genetic relatedness among identified isolates were analyzed using the PFGE method as described previously (31). The similarity index was calculated with the Dice coefficient and dendrogram constructed by the UPGMA (unweighted pair group method using average linkages) algorithm using GelCompar II software (Applied Maths, Belgium).

### PCR amplification and sequencing of chromosomal genes involved in colistin resistance.

The genes *pmrA*, *pmrB*, and *pmrD*, which may be involved in chromosomal colistin resistance were amplified and sequenced. Sequences were then compared to E. coli MG1655 and eight colistin-susceptible clinical E. coli strains (ECS01 to ECS08) to exclude possible synonymous polymorphisms (18). Sequence comparisons were analyzed on the National Center for Biotechnology Information (NCBI) website (www.ncbi.nlm.nih.gov) using the Basic Local Alignment Search Tool (BLAST).

PROVEAN and SIFT software were later used to predict whether the unique amino acid substitutions in PmrA, PmrB, and PmrD from colistin-resistant E. coli strains would affect the function of these proteins.

### Construction of *pmrB* and *pmrA* deletion mutants.

A *pmrA* deletion mutant of MG1655 was generated using plasmid-based gene knockout methods with the pUT-KB plasmid, as previously described, with some modifications (32). Using E. coli MG1655 as a template, two PCR products were created with primer sets KOpmrA_F-1/KOpmrA_R-1 and KOpmrA_F-2/KOpmrA_R-2. The two gel-purified PCR products contained complementary ends and were mixed and amplified using primers KOpmrA_F-1 and KOpmrA_R-2 to create a 693-bp deletion in the *pmrA* gene via overlap PCR. The resulting gel-purified PCR products with 15-bp homologous sequences on both ends of PfoI-digested linear plasmid pUT-KB were cloned into a digested linear pUT-KB plasmid with the In-Fusion HD cloning kit (TaKaRa Bio, Japan), according to the manufacturer’s instructions. Thereafter, the plasmid pUT-KB-KO*pmrA* was constructed. For homologous recombination, pUT-KB-KO*pmrA* was transformed into E. coli MG1655 via electroporation. The transconjugants were screened on Mueller-Hinton agar supplemented with kanamycin (50 mg/liter) and colistin (4 mg/liter). After a single crossover, the kanamycin-resistant transconjugants were selected. Thereafter, the selected transconjugants were incubated for 6 h in 20 ml of brain heart infusion (BHI) broth in the absence of kanamycin at 25°C and then spread onto Luria-Bertani agar containing 10% sucrose. After the double-crossover events, sucrose-resistant and kanamycin-sensitive colonies were selected and the deletion of *pmrA* was confirmed by PCR. The *pmrB* deletion mutants of MG1655 were constructed in an analogous manner, except the primer sets KO*pmrB*_F-1/KO*pmrB*_R-1 and KO*pmrB*_F-2/KO*pmrB*_R-2 were used. The constructs are referred to as MG1655_Δ*pmrA* and MG1655_Δ*pmrB*, respectively.

### Complementation of E. coli MG1655_Δ*pmrA*, E. coli MG1655_Δ*pmrB*, and colistin-resistant E. coli strains.

The amplified *pmrA* and *pmrB* genes from the respective E. coli strains were directly inserted into the pCRII-TOPO TA vector, according to the manufacturer’s protocol (Invitrogen, USA). Since the pCRII-TOPO TA vector is a high-copy-number plasmid that may lead to overexpressed cloned genes, which might constitute a bias in our experiments, wild-type *pmrA* or *pmrB* genes from MG1655 were directly inserted into the pCRII-TOPO TA vector as controls in the complementation experiments. The resulting plasmids were separately transformed into identified colistin-resistant strains, E. coli MG1655_Δ*pmrA* or MG1655_Δ*pmrB* via electroporation. The transformants were selected by overnight incubation at 37°C on kanamycin (50 mg/liter)-supplemented Mueller-Hinton agar, and the presence of the cloned gene was verified via PCR sequencing. The designations of all E. coli transformants are shown in Table S1.

### Real-time RT-PCR.

To evaluate the association between upregulation of the *pmrBCADTEF/pmrCAB* operon and PmrAB TCS, expression levels of the genes *pmrK* and *pmrC*, representative of the *pmrHFIJKLM* and *pmrCAB* operon and *pmrAB*, were estimated. In addition, expression levels of the regulatory gene *pmrD*, which has been reported to be associated with colistin resistance, were also measured (13). Bacterial RNA and cDNA from all tested strains were obtained using an RNeasy minikit (Qiagen, USA) and Prime Script RT master mix (TaKaRa, Japan), according to the manufacturers’ instructions. All measured genes were estimated using SYBR green PCR master mix (Thermo Fisher Scientific, USA). The *gapA* gene served as an endogenous reference for normalizing the expression levels. Data were calibrated against the baseline expression level of E. coli MG1655, and fold change in expression was calculated using the comparative threshold cycle method (33). Data are expressed as means and standard deviations from four independent experiments. A Wilcoxon rank sum test was used for statistical analysis. A *P* value of <0.05 was considered statistically significant.

### Data availability.

The complete nucleotide sequences of the mutated *pmrA* obtained from the colistin-resistant isolates TSAREC01, TSAREC04, and TSAREC41 have been deposited in the GenBank nucleotide database under accession numbers MT586093, MT597414, and MT597415, respectively. The nucleotide sequences of the mutated *pmrB* from the colistin-resistant isolates TSAREC02, TSAREC03, TSAREC05, TSAREC06, TSAREC07, TSAREC08, TSAREC10, and TSAREC37 have been deposited in the GenBank nucleotide database as MT597406, MT597407, MT597408, MT597409, MT597413, MT597410, MT597411, and MT597412, respectively.
