# Erratum for Wang et al., “A Resistance Mechanism in Non-*mcr* Colistin-Resistant Escherichia coli in Taiwan: R81H Substitution in PmrA Is an Independent Factor Contributing to Colistin Resistance”

**DOI:** 10.1128/spectrum.02755-21

**Published:** 2022-02-02

**Authors:** Ching-Hsun Wang, L. Kristopher Siu, Feng-Yee Chang, Sheng-Kang Chiu, Jung-Chung Lin

**Affiliations:** a Graduate Institute of Medical Sciences, National Defense Medical Center, Taipei, Taiwan; b Institute of Infectious Diseases and Vaccinology, National Health Research Institutes, Zhunan, Miaoli, Taiwan; c Division of Infectious Diseases and Tropical Medicine, Department of Internal Medicine, Tri-Service General Hospital, National Defense Medical Center, Taipei, Taiwan

## ERRATUM

Volume 9, no. 1, e00022-21, 2021, https://doi.org/10.1128/Spectrum.00022-21. Page 1: The affiliations should appear as given in this Erratum.

