# Supplementary material for: A Resistance Mechanism in Non-mcr Colistin-Resistant Escherichia coli in Taiwan: R81H Substitution in PmrA Is an Independent Factor Contributing to Colistin Resistance
Source: Microbiol Spectr. 2021 Jul 14;9(1):10.1128/spectrum.00022-21. doi: 10.1128/spectrum.00022-21 (PMC8552686; doi:10.1128/spectrum.00022-21)
Supplement: SUPPLEMENTAL FILE 1 — Supplemental material. Download SPECTRUM00022-21_Supp_1_seq4.pdf, PDF file, 0.2 MB [file spectrum00022-21_supp_1_seq4.pdf]

Table S1. Strains used in this study.

Strain	Relevant characteristic(s)	Source / Reference
MG1655	Wild type	ATCC
ATCC 25922	Wild type	ATCC
J53	Resistant to sodium azide used as a conjugation recipient	(1)
TSAREC01	Clinical isolate with colistin resistance	NIHH
TSAREC02	Clinical isolate with colistin resistance	NIHH
TSAREC03	Clinical isolate with colistin resistance	NIHH
TSAREC04	Clinical isolate with colistin resistance	NIHH
TSAREC05	Clinical isolate with colistin resistance	NIHH
TSAREC06	Clinical isolate with colistin resistance	NIHH
TSAREC07	Clinical isolate with colistin resistance	NIHH
TSAREC08	Clinical isolate with colistin resistance	NIHH
TSAREC10	Clinical isolate with colistin resistance	NIHH
TSAREC37	Clinical isolate with colistin resistance	NIHH
TSAREC41	Clinical isolate with colistin resistance	NIHH
EC909	Colistin resistant isolate with *mcr*-1	(2)
ECS01	Clinical isolate susceptible to colistin, novel ST	(3)
ECS02	Clinical isolate susceptible to colistin, ST73	(3)
ECS03	Clinical isolate susceptible to colistin, ST131	(3)
ECS04	Clinical isolate susceptible to colistin, ST69	(3)
ECS05	Clinical isolate susceptible to colistin, ST38	(3)
ECS06	Clinical isolate susceptible to colistin, ST73	(3)
ECS07	Clinical isolate susceptible to colistin, ST1193	(3)
ECS08	Clinical isolate susceptible to colistin, ST131	(3)
MG1655_Δ*pmrA*	538-bp deletion in the *pmrA* locus of MG1655	This study
MG1655_Δ*pmrB*	693-bp deletion in the *pmrB* locus of MG1655	This study
MG1655_Δ*pmrA* (pCRII-TOPO*pmrA*^{MG1655WT})	MG1655_Δ*pmrA* complemented with pCRII-TOPO*pmrA*^{MG1655WT}	This study
MG1655_Δ*pmrB* (pCRII-TOPO*pmrB*^{MG1655WT})	MG1655_Δ*pmrB* complemented with pCRII-TOPO*pmrB*^{MG1655WT}	This study
MG1655_Δ*pmrA* (pCRII-TOPO*pmrA*^{g242a})	MG1655_Δ*pmrA* complemented with pCRII-TOPO*pmrA*^{g242a}	This study
MG1655_Δ*pmrB* (pCRII-TOPO*pmrB*$^{g616a, t618g, t664c}$)	MG1655_Δ*pmrB* complemented with pCRII-TOPO*pmrB*$^{g616a, t618g, t664c}$	This study
MG1655_Δ*pmrB* (pCRII-TOPO*pmrB*$^{g3c, t41c, c532t, c704a}$)	MG1655_Δ*pmrB* complemented with pCRII-TOPO*pmrB*$^{g3c, t41c, c532t, c704a}$	This study
MG1655_Δ*pmrB* (pCRII-TOPO*pmrB*^{c281t})	MG1655_Δ*pmrB* complemented with pCRII-TOPO*pmrB*^{c281t}	This study

MG1655_Δ*pmrB* (pCRII-TOPO*pmrB*^{g56a})	MG1655_Δ*pmrB* complemented with pCRII-TOPO*pmrB*^{g56a}	This study
MG1655_Δ*pmrB* (pCRII-TOPO*pmrB*^{t581c})	MG1655_Δ*pmrB* complemented with pCRII-TOPO*pmrB*^{t581c}	This study
MG1655_Δ*pmrB* (pCRII-TOPO*pmrB*^{t293g})	MG1655_Δ*pmrB* complemented with pCRII-TOPO*pmrB*^{t293g}	This study
MG1655_Δpmr*B* (pCRII-TOPO*pmrB*^{t80g})	MG1655_Δ*pmrB* complemented with pCRII-TOPO*pmrB*^{t80g}	This study
TSAREC02 (pCRII-TOPO*pmrB*^{MG1655WT})	TSAREC02 complemented with pCRII-TOPO*pmrB*^{MG1655WT}	This study
TSAREC03 (pCRII-TOPO*pmrB*^{MG1655WT})	TSAREC03 complemented with pCRII-TOPO*pmrB*^{MG1655WT}	This study
TSAREC05 (pCRII-TOPO*pmrB*^{MG1655WT})	TSAREC05 complemented with pCRII-TOPO*pmrB*^{MG1655WT}	This study
TSAREC06 (pCRII-TOPO*pmrB*^{MG1655WT})	TSAREC06 complemented with pCRII-TOPO*pmrB*^{MG1655WT}	This study
TSAREC08 (pCRII-TOPO*pmrB*^{MG1655WT})	TSAREC08 complemented with pCRII-TOPO*pmrB*^{MG1655WT}	This study
TSAREC10 (pCRII-TOPO*pmrB*^{MG1655WT})	TSAREC10 complemented with pCRII-TOPO*pmrB*^{MG1655WT}	This study
TSAREC37 (pCRII-TOPO*pmrB*^{MG1655WT})	TSAREC37 complemented with pCRII-TOPO*pmrB*^{MG1655WT}	This study

Table S2. Plasmid used in this study.

Plasmid	Relevant characteristic(s)	Source / Reference
pCRII-TOPO	TOPO cloning vector; Amp^r Kan^r	Invitrogen
pUT-KB	Suicide vector; Kan^r	(4)
pCRII-TOPO*pmrA*^{MG1655WT}	The WT-*pmrA* gene from *E. coli* MG1655 cloned into pCRII-TOPO	This study
pCRII-TOPO*pmrB*^{MG1655WT}	The WT-*pmrB* gene from *E. coli* MG1655 cloned into pCRII-TOPO	This study
pCRII-TOPO*pmrA*^{g242a}	The *pmrA* gene from *E. coli* TSAREC01 cloned into pCRII-TOPO	This study
pCRII-TOPO*pmrB*$^{g616a, t618g, t664c}$	The *pmrB* gene from *E. coli* TSAREC02 cloned into pCRII-TOPO	This study
pCRII-TOPO*pmrB*$^{g3c, t41c, c532t, c704a}$	The *pmrB* gene from *E. coli* TSAREC03 cloned into pCRII-TOPO	This study
pCRII-TOPO*pmrB*^{c281t}	The *pmrB* gene from *E. coli* TSAREC05 cloned into pCRII-TOPO	This study
pCRII-TOPO*pmrB*^{g56a}	The *pmrB* gene from *E. coli* TSAREC06 cloned into pCRII-TOPO	This study
pCRII-TOPO*pmrB*^{t581c}	The *pmrB* gene from *E. coli* TSAREC08 cloned into pCRII-TOPO	This study
pCRII-TOPO*pmrB*^{t293g}	The *pmrB* gene from *E. coli* TSAREC10 cloned into pCRII-TOPO	This study
pCRII-TOPO*pmrB*^{t80g}	The *pmrB* gene from *E. coli* TSAREC37 cloned into pCRII-TOPO	This study
pUT-KB-KO*pmrA*	2,334-bp fragment with a 538-bp deletion in *pmrA* from *E. coli* MG 1655 cloned into pUT-KB	This study
pUT-KB-KO*pmrB*	2,886-bp fragment with a 693-bp deletion in *pmrB* from *E. coli* MG1655 cloned into pUT-KB	This study

Amp^r, resistance to ampicillin; Kan^r, resistance to kanamycin.

Table S3. Primers used in this study

Aplication and Primer name	Sequence (5′-3′)	Reference
**PCR and DNA sequencing**		
mcr-1-F	AGTCCGTTTGTTCTTGTGGC	(5)
mcr-1-R	AGATCCTTGGTCTCGGCTTG	
mcr-2-F	TCATTGTGGTTGTCCTTTTCTG	(5)
mcr-2-R	TCTAGCCCGACAAGCATACC	
mcr-3-F	AAATAAAAATTGTTCCGCTTATG	(5)
mcr-3-R	AATGGAGATCCCCGTTTTT	
mcr-4-F	TCACTTTCATCACTGCGTTG	(5)
mcr-4-R	TTGGTCCATGACTACCAATG	
mcr-5-F	ATGCGGTTGTCTGCATTTATC	(5)
mcr-5-R	TCATTGTGGTTGTCCTTTTCTG	
mcr-6-F	AGCTATGTCAATCCCGTGAT	(6)
mcr-6-R	ATTGGCTAGGTTGTCAATC	
mcr-7-F	GCCCTTCTTTTCGTTGTT	(6)
mcr-7-R	GGTTGGTCTCTTTCTCGT	
mcr-8-F	TCAACAATTCTACAAAGCGTG	(6)
mcr-8-R	AATGCTGCGCGAATGAAG	
mcr-9-F	TTCCCTTTGTTCTGGTTG	(6)
mcr-9-R	GCAGGTAATAAGTCGGTC	
pmrA-F	TGCTGTGGCTGTCGGA	(7)
pmrA-R	AATCTGCTCGGTACTTTCATG	
pmrB-F	CCAACACCCTGGAAGTGC	(7)
pmrB-R	TGATGAATAAGCTGAAACGGA	
pmrD-F	GATGTGAAAACTCTTAGCAAAC	This study
pmrD-R	TGATGCTGATTTTCCTGC	
**Mutagenesis**		
KO*pmrA*_F-1	ACATGCAGCTCCCGGACTACCCGCGTGAAACTAA [a]	This study
KO*pmrA*_R-1	CAATAACAGCGTATCGTCTTCAACAATCAG [b]	
KO*pmrA*_F-2	CTGATTGTTGAAGACGATACGCTGTTATTGATATCCACAATCTGCGCG [b]	This study
KO*pmrA*_R-2	GTGACCGTCTCCGGGAGTTTCGCAATAACATCCG [a]	
KO*pmrB*_F-1	ACATGCAGCTCCCGGACCTGTACCAAAGAGCAACTGGTGAAC [a]	This study
KO*pmrB*_R-1	GCATCAGATTCAATTAGTTTTCCTCATTCG [b]	

KO*pmrB*_F-2	CGAATGAGGAAAACTAATTGAATCTGATGCAGCAAACCCTGCTATTGCC[b]	This study
KO*pmrB*_R-2	GTGACCGTCTCCGGGACGTCCGTTTGTGCTTCTTGGTAGTG [a]	
**Real-time RT-PCR**		
pmrK-F	TGCGGAAATCAGTCGAGAAATGC	(8)
pmrK-R	CGAAATAACGTAGCCCTAACAGATGG	
pmrB-F	TCCCCTCGTATGACGAACTC	(9)
pmrB-R	TCATAATGTTGCTGCCTTGC	
pmrC-F	CGACAACACGCTGGTTTACGTCGA	(10)
pmrC-R	ACCGTGGTCAGAAAGATAAACCAGGC	
pmrD-F	ATGGAATGGCTGGTCAAAAA	(11)
pmrD-R	CATTCTGCAAAGGCGAGAGT	
pmrA-F	GGGCGGTGAAGAGTTGATT	(11)
pmrA-R	TTGGTCGAGGGTTCATTGTC	
gapA-F	CGACAAATATGCTGGCCAGG	(8)
gapA -R	GTAGTAGCGTGAACGGTGGT	

[a] The underlined sequences are corresponding to the overlapping region of PfoI-digested linear plasmid pUT-KB for In-Fusion cloning.

[b] The underlined sequences are complementary to the noncontiguous sequence and were used to introduce internal deletions by overlap PCR.

Reference

1. Coetzee JN, Datta N, Hedges RW. 1972. R factors from Proteus rettgeri. J Gen Microbiol 72:543–552.
2. Ching-Hsun Wang, Jung-Chung Lin, Ching-Mei Yu, Rui-Xin Wu. 2020. Emergence of multiple drug-resistant Escherichia coli harboring mcr-1 in immunocompetent patients from the community. J Microbiol Immunol Infect. 53: 663-664.
3. Wang CH, Siu LK, Chang FY, Tsai YK, Lin YT, Chiu SK, Huang LY, Lin JC. 2020. A novel deletion mutation in pmrB contributes to concurrent colistin resistance in carbapenem-resistant Escherichia coli sequence Type 405 of clinical origin. Antimicrob Agents Chemother 64(6):e00220-20.
4. Yu-Kuo Tsai, Ci-Hong Liou, Jung-Chung Lin, Ling Ma, Chang-Phone, Fung, Feng-Yee Chang, et al. 2014. A suitable streptomycin-resistant mutant for constructing unmarked in-frame gene deletions using rpsL as a counter-selection marker. PLoS One. 30; 9: e109258. doi: 10.1371/journal.pone.0109258. eCollection 2014.
5. Ana Rita Rebelo, Valeria Bortolaia, Jette S Kjeldgaard, Susanne K Pedersen, Pimlapas Leekitcharoenphon, Inge M Hansen, et al. 2018. Multiplex PCR for detection of plasmid-mediated colistin resistance determinants, mcr-1, mcr-2, mcr-3, mcr-4 and mcr-5 for surveillance purposes. Euro Surveill. 23:17-00672. doi: 10.2807/1560-7917.ES.2018.23.6.17-00672.
6. Maria Borowiak, Beatrice Baumann, Jennie Fischer, Katharina Thomas, Carlus Deneke, Jens Andre Hammerl, et al. 2020. Development of a novel mcr-6 to mcr-9 multiplex PCR and assessment of mcr-1 to mcr-9 occurrence in colistin-resistant Salmonella enterica isolates from environment, feed, animals and food (2011–2018) in Germany. Front Microbiol;11:80. doi: 10.3389/fmicb.2020.00080. eCollection 2020.
7. Jingjing Quan, Xi Li, Yan Chen, Yan Jiang, Zhihui Zhou, Huichuan Zhang, et al. 2017. Prevalence of mcr-1 in Escherichia coli and Klebsiella pneumoniae recovered from bloodstream infections in China: a multicentre longitudinal study. Lancet Infect Dis. 17:400–410.
8. Antonio Cannatelli, Tommaso Giani, Noemi Aiezza, Vincenzo Di Pilato, Luigi Principe, Francesco Luzzaro, et al. 2017. An allelic variant of the PmrB sensor kinase responsible for colistin resistance in an Escherichia coli strain of clinical origin. Sci Rep. 11; 7:5071. doi: 10.1038/s41598-017-05167-6.
9. Minh-Duy Phan, Nguyen Thi Khanh Nhu, Maud E S Achard, Brian M Forde, Kar Wai Hong, Teik Min Chong, et al. 2017. Modifications in the pmrB gene are the primary

mechanism for the development of chromosomally encoded resistance to polymyxins in uropathogenic Escherichia coli. J Antimicrob Chemother. 72: 2729–2736. doi: 10.1093/jac/dkx204.

10. Toyotaka Sato, Tsukasa Shiraishi, Yoshiki Hiyama, Hiroyuki Honda, Masaaki Shinagawa, Masaru Usui, etc. 2018. Contribution of novel amino acid alterations in PmrA or PmrB to colistin resistance in mcr-negative Escherichia coli clinical isolates, including major multidrug-resistant lineages O25b:H4-ST131- H 30Rx and Non-x. 2018. Antimicrob Agents Chemother 62:e00864-18. doi: 10.1128/AAC.00864-18.
11. Erica J Rubin, Carmen M Herrera, Alexander A Crofts, M Stephen Trent. 2015. PmrD is required for modifications to Escherichia coli endotoxin that promote antimicrobial resistance. Antimicrob Agents Chemother. 59:2051–2061. doi: 10.1128/AAC.05052-14.
